# Further advances in the diagnosis and treatment of Leber’s Hereditary Optic Neuropathy – a review

**DOI:** 10.22336/rjo.2022.4

**Published:** 2022

**Authors:** Bogdana Tăbăcaru, Horia Tudor Stanca

**Affiliations:** * Clinical Department of Ophthalmology, “Prof. Dr. Agrippa Ionescu” Emergency Hospital, Bucharest, Romania; ** Clinic of Ophthalmology, “Carol Davila” Medicine and Pharmacy University, Bucharest, Romania

**Keywords:** atypical mutations, gene therapy, idebenone, recessive inheritance

## Abstract

Leber’s Hereditary Optic Neuropathy (LHON), is one of the most frequent mitochondrial diseases characterized by Retinal Ganglion Cells degeneration. Pathogenic gene mutations in LHON induces mitochondrial impairment, which in turn leads to insufficient mitochondrial ATP production. The pathologic hallmark of the disease is primary degeneration of retinal ganglion cells, which results in optic nerve atrophy.

The paper reviews some of the recent advances in the understanding of LHON: new genetics discoveries and novel therapeutic approaches.

## Introduction

Leber’s Hereditary Optic Neuropathy (LHON), the most frequent mitochondrial disease, was described as a clinical entity by Theodor Leber in 1871 [**1**], but was first noted a few years before, by Von Graefe, in 1858 [**1**]. The first point mutation was discovered by Wallance et al., one century later, in 1988, in the mitochondrial DNA (mtDNA) (m.11778G>A in MT-ND4) [**2**] and further other two-point mutations were described (m.3460G>A in MT-ND1 and m.14484T>C in MT-ND6) [**1**,**3**-**5**]. These three mtDNA mutations are responsible for approximately 90% of LHON cases (mtLHON) [**1**,**3**-**5**]. Another 5% of LHON cases are produced by rare mtDNA mutations, the so-called „exotic mutations”, including variants associated with systemic pathology (e.g., MELAS and Leigh syndrome) [**1**,**6**]. All these mtDNA mutations disrupt subunits of mitochondrial complex I, impairing the electron transfer and the ATP synthesis [**1**].

## Advances in LHON Genetics

Stenon et al. recently described an autosomal recessive model of inheritance for LHON (arLHON), resolving the problem of undiagnosed patients with LHON manifestations and no proof of pathogenic mtDNA mutations [**1**]. The mutation was identified in a nuclear encoded gene, DNAJC30 [**1**]. The DNAJC30 proved to be a chaperone protein needed for the efficient complex I function [**1**]. In arLHON, all characteristics of mtLHON were present, including: incomplete penetrance (not all family members were affected), male predominance and significant idebenone responsivity [**1**].

High-resolution restriction-endonuclease analysis was performed in LHON patients to define the phylogenetic relationships between the mtDNA haplotypes and the LHON mutations [**7**,**8**]. Except for some few pairs and triplets of identical haplotypes that were observed, the large majority of the LHON mutations were due to independent mutational events [**8**]. The analysis showed that the primary mutations 11778, 3460, and 14484 were recent and were due to multiple mutational events [**7**]. However, there are other putative secondary/ intermediate LHON mutations (e.g., 4216, 4917, 13708, 15257 and 15812) that are ancient polymorphisms and are associated in specific combinations, especially in haplotype group J that is European-specific [**7**]. The 11778 and 14484 primary mutations also showed a strong preferential association with haplogroup J, this meaning that one ancient combination of haplogroup J-specific mutations increases both the penetrance of the two primary mutations 11778 and 14484 and the risk of disease expression [**7**].

A key issue in LHON molecular diagnosis is the tissue used for sequence analysis. Some mtDNA mutations are counterselected in a fast turnover tissue like the cells from the blood sample, and disappear in an age-related fashion. Therefore, different tissues should better be collected for the LHON molecular diagnosis. Except for the blood, the total DNA may be extracted from tissues including skeletal muscle, buccal epithelia, and urinary sediments [**9**]. The skeletal muscle biopsy and the urinary sediments are the gold standard, but the skeletal muscle biopsy is invasive and might not be justified in some cases.

A rare possibility is to demonstrate two primary mutations in the same patient [**10**]. in 2016, Catarino et al. reported a LHON family with multiple patients harboring two primary LHON mutations, m.11778G>A homoplasmic and m.14484T>C heteroplasmic [**10**]. Some atypical findings were registered in this family [**10**]. The male-to-female ratio was unusually low as all three affected family members were females [**10**]. The index patient had a very late onset of symptoms (at 75 years) and low visual acuity, but her two daughters had both onset in childhood (6 and 9 years), with moderate to mild visual impairment [**10**]. A higher degree of heteroplasmy of the m.14484T>C mutation was found to correlate with an earlier age at onset in this family [**10**]. 

When considering the children with LHON or patients with early onset of the disease, the specific acute/ subacute phase may be absent or overlooked, as for a child it is difficult to assess the moment when visual acuity decreases. When examining pediatric patients, the ophthalmologist may find optic nerve atrophy from the first presentation of the patient. Therefore, differential diagnosis between pediatric LHON and Dominant Optic Atrophy (DOA) may be challenging. The diagnosis of DOA can be ruled out by genetic analyses, OPA1 mutations covering 70% of DOA cases [**11**]. OPA1 is a nuclear gene that regulates the mitochondrial fusion and dynamics and is involved in the oxidative phosphorylation [**12**,**13**]. Nowadays, other genes involved in DOA for patients negative for OPA1 mutations have been described [**11**]. AFG3L2 and SPG7 genes showed to have a crucial role in the maintenance of the optic nerve physiology and must be screened when suspecting DOA patients [**14**].

## Therapeutic options in LHON 

The Retinal Ganglion Cells (RGCs) degeneration is the common pathogenic event of LHON, DOA and others Hereditary Optic Neuropathies [**12**]. Therapeutic strategies are now limited to LHON, but hopefully they will also be offered soon in other mitochondrial optic neuropathies [**12**]. 

In order to avoid blocking the light to reach to the retinal pigment epithelium, in the macular region and particularly in the papillomacular bundle, the RGCs have unmyelinated narrower caliber axons [**12**,**15**-**20**]. This anatomical particularity is associated with an increased energy requirement, when the saltatory conduction of action potentials is absent. Therefore, the macular RGCs axons demonstrated to have mitochondrial clustering and a special sensitivity to energy depletion and reactive oxidative species (ROS) accumulation as it happens in mitochondrial dysfunctions [**12**,**15**-**24**]. The optic nerve has normal myelinated axonal structure posterior to the lamina cribrosa [**12**,**19**].

Therapeutic options for Hereditary Optic Neuropathies and innovative drugs acting on different molecular pathways are nowadays under study [**12**]. These include: antioxidants, anti-apoptotic drugs, activators of mitobiogenesis and gene therapy [**12**].

Idebenone (Raxone®) was the first molecule approved for LHON in Europe in 2015 and due to the low prevalence of the LHON it is an orphan drug [**12**]. Idebenone acts as a strong antioxidant that restores the ATP synthesis at mitochondrial level in LHON patients by direct electrons transfer to complex III of the electron transport chain, thereby bypassing the unfunctional complex I [**19**,**25**]. The efficacy and safety of idebenone was assessed by Klopstock et al. in 2011 in the RHODOS study [**12**,**19**,**26**-**28**]. The experts’ consensus of idebenone recommends that the treatment should start in the first year from visual loss onset (in the acute stage), as early as possible, the administration of the idebenone dose of 900 mg/ day being given into three daily doses of 300 mg with meals [**12**,**29**]. Treatment should be given for at least 12 months to assess a therapeutic response, then discontinuation of the drug should be considered after 12 months once a plateau recovery is reached, or no improvements are observed [**12**,**29**]. Two others multicenter studies (LEROS and PAROS) were launched after the idebenone commercial approval, being designed to assess the long-term efficacy and safety of the drug [**12**]. The results are now being processed and will be published soon. Pump et al. recently published new promising results with visual improvement after treating patients in both early chronic phase (1-5 years after clinical onset) and late chronic phase (>5 years) with idebenone [**30**].

Epi-743 (vatiquinone) is an antioxidant drug acting on oxidoreductase enzymes [**12**]. In 2012, Sadun et at. demonstrated the arrest of the disease progression and improvement of visual acuity in four of five patients with LHON treated with Epi-743, three times daily, with an oral dose of 100-400 mg per dose, for 90 days [**31**]. Since then, the Epi-743 was not used any more in LHON patients [**31**]. In 2018, Zesiewicz et al. demonstrated the Epi-743 efficacy in the improvement of both the neurological function and the disease progression in 63 patients with Friedreich’s ataxia, but, in this study, the Epi-743 failed to demonstrate any significant visual benefit in the two-thirds of the treated patients who associated optic atrophy [**31**,**32**]. 

Elamipretide (MTP-131) is a molecule that increases the ATP synthesis and reduces the ROS production, regardless of the type of mitochondrial abnormality [**12**,**33**]. Elamipretide is a Szeto-Schiller antioxidant peptide that targets cardiolipin selectively in the inner mitochondrial membrane, this way preventing conversion of cytochrome c into a peroxidase, preserving its function as simple electron carrier, and promoting OXPHOS functioning [**12**]. Sadun et al. conducted a Phase II Clinical Study (ReSIGHT) to evaluate the safety, tolerability, and efficacy of the Topical Ophthalmic Solution of Elamipretide for 12 patients with LHON [**12**]. The results are not yet published, but a press release announced promising results [**12**].

Cell degradation and self-destruction of the mitochondria are activated by caspases that are specific enzymes in the apoptosis cascade [**12**]. So far, only the Cyclosporine A, an inhibitor of the opening of the mitochondrial permeability transition pore, has been inefficiently tested in five LHON patients [**12**,**34**]. Other molecules inhibiting different apoptotic events may represent future therapeutic agents for hereditary optic neuropathies in future [**12**].

Photobiomodulation by near-infrared light-emitting diode arrays (NIR-LED) is a promising therapy in LHON as it was demonstrated that it attenuates the optic nerve degeneration after acute mitochondrial injuries with different toxins in a rat model. Human research is necessary but the only study set by now failed to recruit enough patients [**12**,**35**].

Gene therapy is the goal in hereditary optic neuropathies treatment [**12**]. To date, there are several phase I, II and III studies for LHON gene therapy [**12**]. Promising results were obtained on LHON patients with m.11778G>A mitochondrial DNA mutation [**12**]. The gene therapy consisted in a wild-type of ND4 subunit that was packaged into an adeno-associated-virus and injected into the vitreous [**12**]. RESCUE and REVERSE are two randomized, double-masked, sham-controlled, multi-center, phase III clinical studies in which an intravitreal administration of rAAV2/ 2-ND4 (GS010) was delivered in one eye and an intravitreal administration of sham injection was delivered in the fellow eye [**12**,**36**,**37**]. 37 patients with visual acuity loss duration of 180 days or less were included in the RESCUE study and 39 patients with the onset of the disease between 6 and 12 months were included in the REVERSE study [**12**,**36**,**37**]. In both studies, a statistically significant visual acuity improvement was surprisingly demonstrated both in the GS010-treated eyes and in the sham-treated eyes [**12**,**36**,**37**]. A non-clinical trial on primates suggested a possible retrograde, trans-chiasmatic transit of the vector from the GS010-treated eye to the sham-treated eye as explanation for the unexpected bilateral visual improvement found in the RESCUE and REVERSE studies [**12**]. Recently, in 2019, the enrollment of 98 patients in a new Phase III clinical trial (REFLECT) was completed [**12**,**38**]. The REFLECT study will evaluate the efficacy and safety of bilateral intravitreal injections of GS010 in subjects with 11778 LHON mutation [**12**,**38**].

## Conclusion

Advances in the diagnosis and treatment of LHON are currently in a positive dynamic direction. New pathogenic mechanisms, novel therapeutic molecules and gene therapy are the near future approach in LHON and other mitochondrial optic disorders.

**Conflict of Interest statement**

The authors state no conflict of interest. 

**Acknowledgements**

None.

**Sources of Funding**

None. 

**Disclosures**

None.
